# Supplementary material for: Bright Yellow Luminescence from Mn2+-Doped Metastable Zinc Silicate Nanophosphor with Facile Preparation and Its Practical Application
Source: Nanomaterials (Basel). 2024 Aug 27;14(17):1395. doi: 10.3390/nano14171395 (PMC11397549; doi:10.3390/nano14171395)
Supplement: Supplementary file 1 [file nanomaterials-14-01395-s001.zip › nanomaterials-3149032-supplementary.pdf]

Supporting Information

Bright yellow luminescence from Mn^2-doped metastable zinc silicate nanophosphor with facile preparation and its practical application

Mohammad M. Afandi[1], Sanghun Byeon[1], Taewook Kang[2], Hyeonwoo Kang[1], Jongsu Kim[1,*]

[1]Department of Display Science and Engineering, Pukyong National University, Busan 48513, Republic of Korea

[2]Electric Convergence Materials Division, Optic & Electronic Component Materials Center, Korea Institute of Ceramic and Technology, Jinju 52851, Republic of Korea

Supporting Information

This file includes the list of appendixes and several supplementary images (Figures S1 ~ S5) for a greater understanding of the manuscript.

Figures

Figure S1. Flowchart of the synthesis β-Zn_2SiO_4:Mn^{2+} nanophosphor S-3

Figure S2. Photoluminescence excitation (PLE) and photoluminescence emission (PL) of the as-grown α-Zn_2SiO_4:Mn^{2+} with 5 mol% Mn^{2+} concentration annealed at 900 °C (heating rate of 5 °C/min) for 4 hours S-4

Figure S3. Normalized PLE spectra of the as-grown β-Zn_2SiO_4:Mn^{2+} (BZSM) and transformed α-Zn_2SiO_4:Mn^{2+} monitoring under 575 nm and 525 nm, respectively S-5

Figure S4. PL spectra of the Zn_2SiO_4:Mn^{2+} with 5 mol% Mn^{2+} concentration according to annealing temperatures for 10 min. with Gaussian deconvolution fitting the α- and β-Zn_2SiO_4:Mn^{2+} emissions S-6

Figure S5. PL spectra of the Zn_2SiO_4:Mn^{2+} with 5 mol% Mn^{2+} concentration according to synthesis durations at 800 °C with Gaussian deconvolution fitting the α- and β-Zn_2SiO_4:Mn^{2+} emissions S-7

Appendix

BZSM	β-Zn_2SiO_4:Mn^{2+}
EDS	Energy dispersive X-ray spectroscopy
FE-TEM	field emission transmission electron microscopy
FWHM	Full width at half maximum
λ_{em}	Emission/monitoring wavelength
λ_{ex}	Excitation wavelength
PL	Photoluminescence emission
PLE	Photoluminescence excitation
TEM	transmission electron microscopy
UV	Ultraviolet
Vis	Visible
VUV	Vacuum ultraviolet
X-PDT	X-ray-induced photodynamic therapy
XRD	X-ray diffraction
Zinc silicate	Zn_2SiO_4

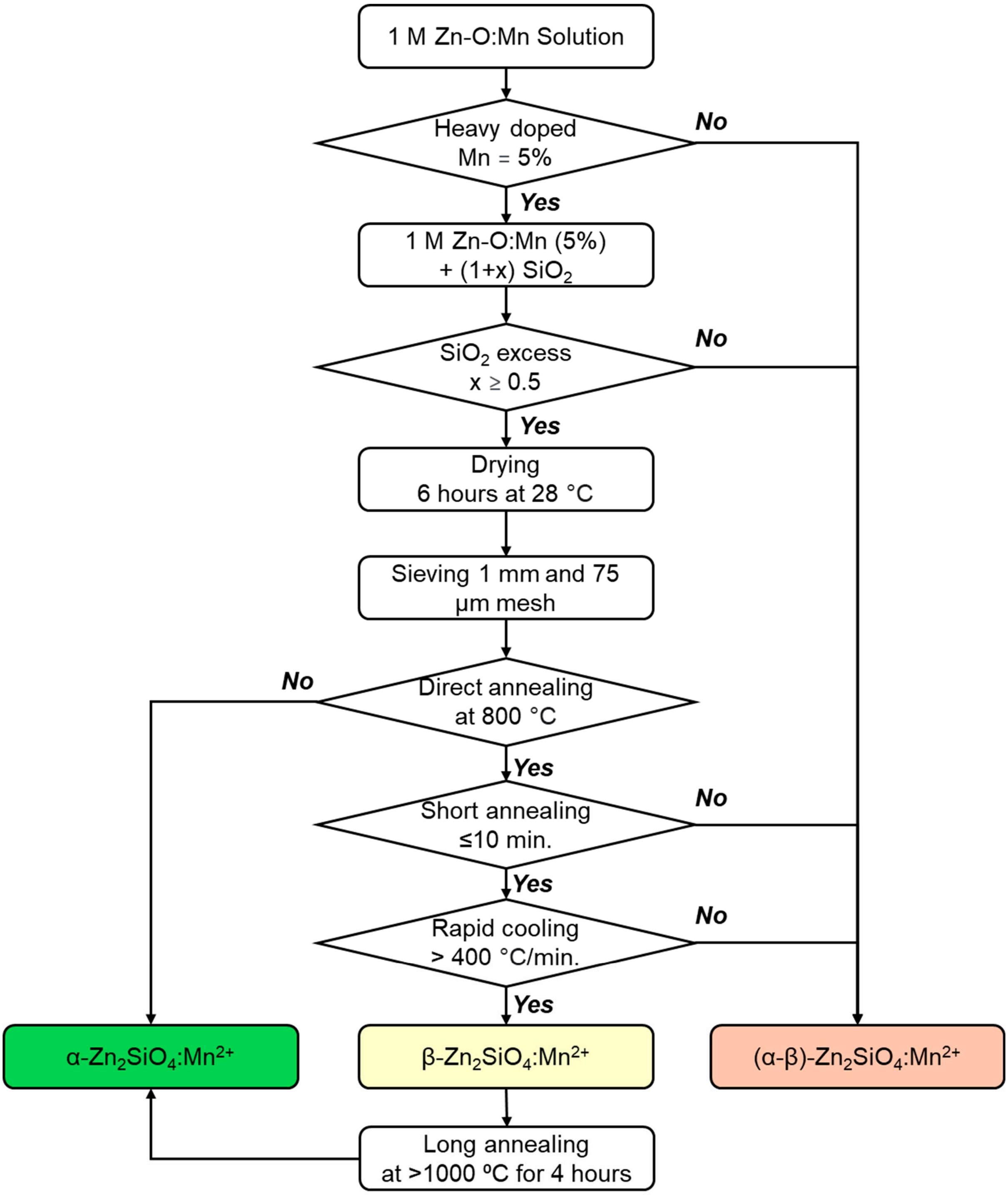

Figure S1. Flowchart of the synthesis β-Zn_2SiO_4:Mn^{2+} nanophosphor.

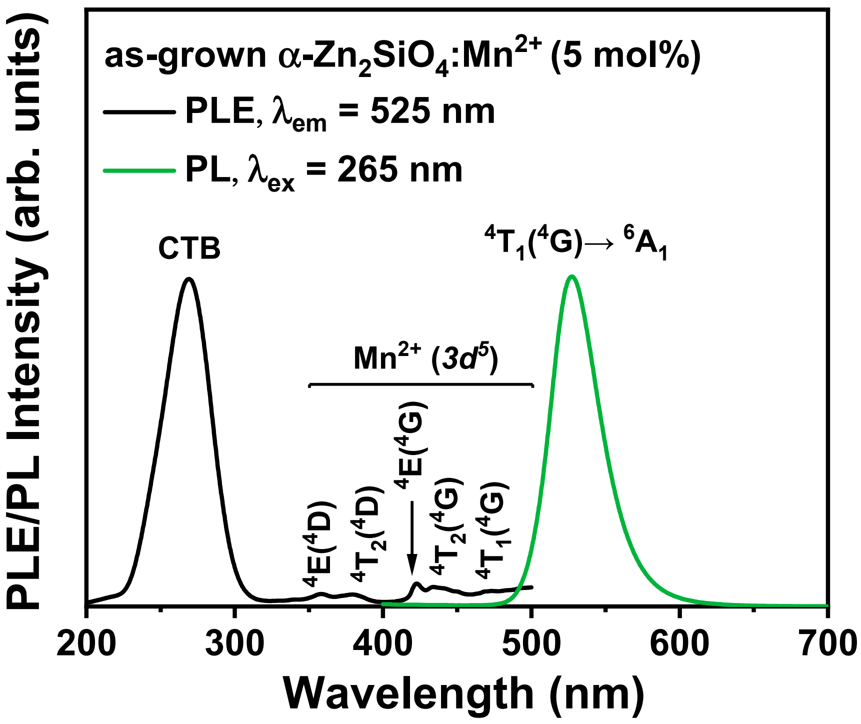

Figure S2. Photoluminescence excitation (PLE) and photoluminescence emission (PL) of the as-grown α-Zn_2SiO_4:Mn^{2+} with 5 mol% Mn^{2+} concentration annealed at 900 °C (heating rate of 5 °C/min) for 4 hours.

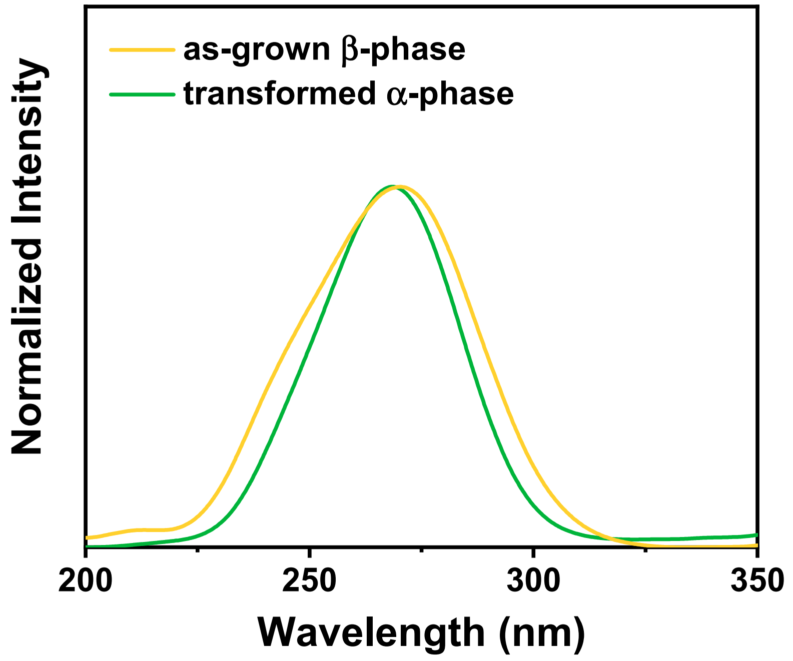

Figure S3. Normalized PLE spectra of the as-grown β-Zn_2SiO_4:Mn^{2+} (BZSM) and transformed α-Zn_2SiO_4:Mn^{2+} monitoring under 575 nm and 525 nm, respectively.

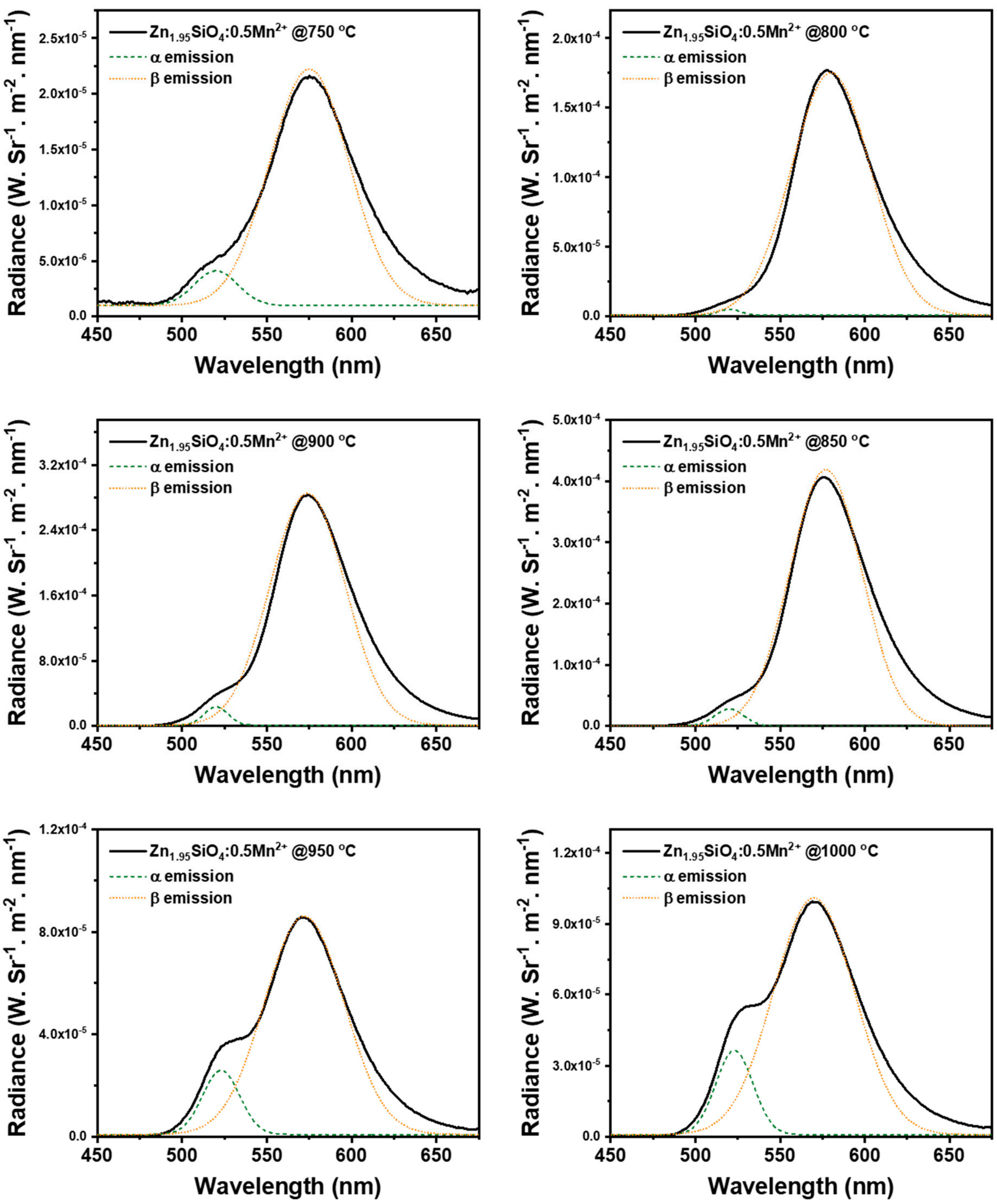

Figure S4. PL spectra of the $Zn_2SiO_4:Mn^{2+}$ with 5 mol% Mn^{2+} concentration according to annealing temperatures for 10 min. with Gaussian deconvolution fitting the α- and β-$Zn_2SiO_4:Mn^{2+}$ emissions.

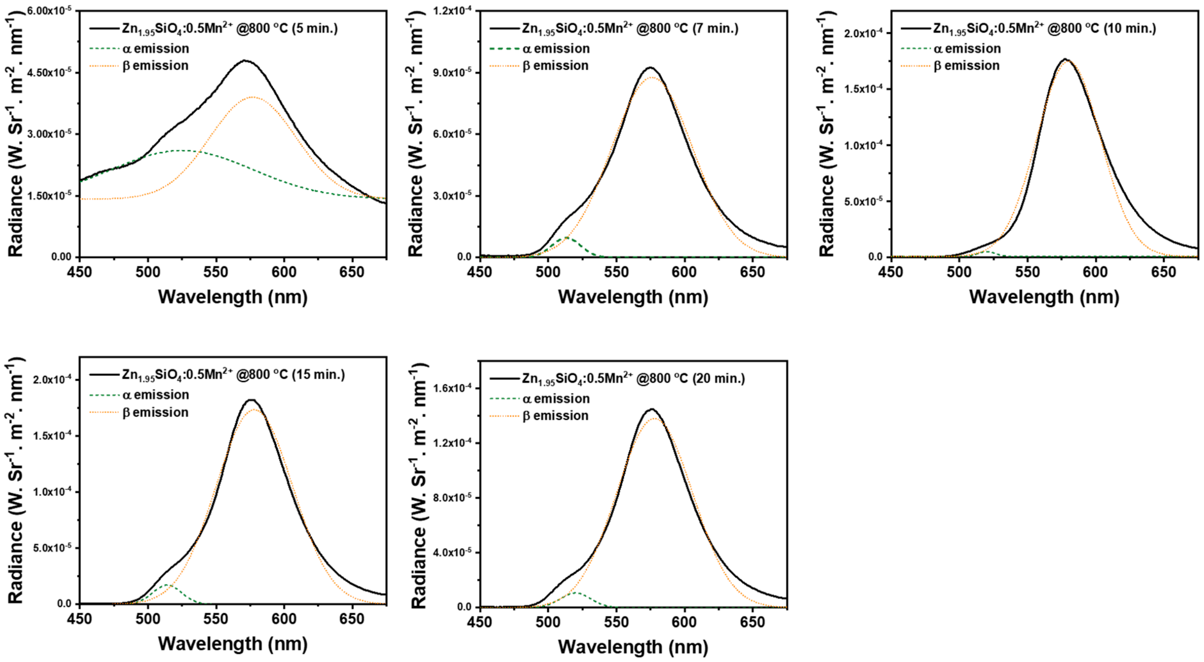

Figure S5. PL spectra of the $Zn_2SiO_4:Mn^{2+}$ with 5 mol% Mn^{2+} concentration according to synthesis durations at 800 °C with Gaussian deconvolution fitting the α- and β-$Zn_2SiO_4:Mn^{2+}$ emissions.
